# Quality of lumbar paraspinal muscles in patients with chronic low back pain and its relationship to pain duration, pain intensity, and quality of life

**DOI:** 10.1007/s00330-024-11236-y

**Published:** 2024-12-07

**Authors:** Sihai Liu, Sandra Reitmaier, Lukas Mödl, Daishui Yang, Tianwei Zhang, Luis Becker, Bernhard Hoehl, Lukas Schönnagel, Torsten Diekhoff, Matthias Pumberger, Hendrik Schmidt

**Affiliations:** 1 Berlin Institute of Health, Julius Wolff Institute for Biomechanics and Musculoskeletal Regeneration, Charité—Universitätsmedizin Berlin, Berlin, Germany; 2 Institute of Biometry and Clinical Epidemiology, Charité—Universitätsmedizin Berlin, Berlin, Germany; 3 Center for Musculoskeletal Surgery, Charité—Universitätsmedizin Berlin, Berlin, Germany; 4 Department for Radiology, Charité—Universitätsmedizin Berlin, Berlin, Germany

**Keywords:** Chronic low back pain, Paraspinal muscles, Magnetic resonance imaging, Quality of life

## Abstract

**Objectives:**

To examine the relationship between the quality of paraspinal muscles and pain intensity, duration, and quality of life in patients with chronic low back pain (cLBP).

**Methods:**

Between January 2022 and December 2023, 119 individuals with no-back pain (no-BP) and 136 cLBP patients were enrolled. Both groups underwent health surveys and magnetic resonance imaging. Cross-sectional area (CSA), functional cross-sectional area (FCSA), and fat infiltration (FI) of multifidus (MF) and erector spinae (ES) were measured. Data were analyzed using multiple linear and binary logistic regression.

**Results:**

Compared to the cLBP group, the no-BP group had smaller CSA influenced by FI of ES at L5/S1 (*p* = 0.01), higher FCSA of ES (*p* < 0.01) at L4/L5, and lower FI of ES and MF at L4/L5 and L5/S1 (*p* < 0.01). CSA, FCSA, and FI showed no significant correlation with cLBP intensity except for the CSA (*p* = 0.02) and FCSA (*p* = 0.03) of the L2/3 MF. Pain duration positively correlated with FI at L2/3, L3/4, and L4/5 of MF and ES (*p* < 0.05) and CSA of the L1/2 MF (*p* = 0.02). CSA (L3/4, L4/5, and L5/S1) and FCSA (L4/5, L5/S1) of MF correlated positively with SF36 scores (*p* < 0.05), while ES muscles did not (*p* > 0.05). FI of MF and ES showed no correlation with SF36 scores.

**Conclusion:**

CSA and FI were significantly higher, and FCSA significantly lower in paraspinal muscles of cLBP patients compared to asymptomatic participants. Increased FI correlated with prolonged cLBP duration, indicating FI and FCSA alterations may play a significant role in cLBP development and duration.

**Key Points:**

***Question*** *What is the relationship between paraspinal muscle quality and cLBP, including its intensity, duration, and impact on quality of life*?

***Findings*** *cLBP patients had increased FI and reduced functional muscle area in paraspinal muscles, with FI correlating with prolonged pain duration*.

***Clinical relevance*** *Understanding the changes in lumbar paraspinal muscles provides insight into cLBP progression, guiding personalized interventions to improve pain management and patient quality of life*.

**Graphical Abstract:**

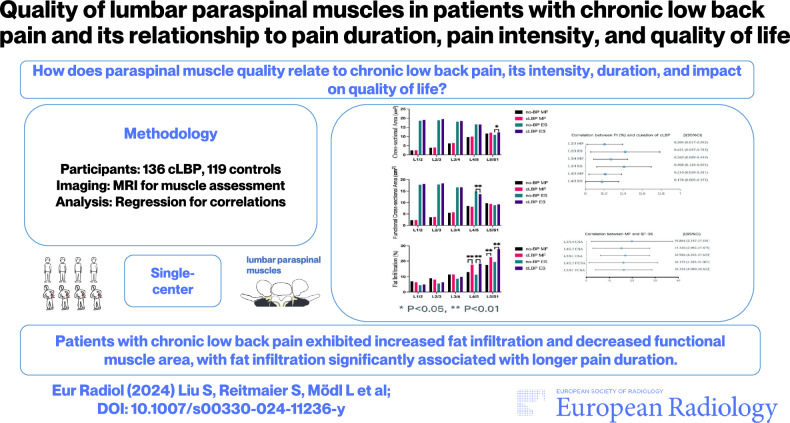

## Introduction

Low back pain is a prevalent condition that affects nearly 80% of individuals at some point in their lives [1, 2]. Epidemiological studies suggest that 5–10% of people develop chronic low back pain (cLBP), which leads to significant treatment costs, a high economic burden due to sickness absence, and individual suffering [3, 4]. Additionally, it is one of the main reasons for healthcare utilization [5]. Even though back pain is increasingly perceived within the context of a biopsychosocial disease model, studies have shown that degeneration of the lumbar spine, which includes the intervertebral disks, lumbar fasciae, facet joints, and paraspinal muscles, may contribute to the developing of LBP [6–10].

Biomechanically, it is well established that the paraspinal muscles, including the multifidus (MF) and erector spinae (ES) muscles, support and stabilize the spine and control spinal movements. However, controversy exists regarding the potential relationship between the quality of lumbar paraspinal muscles and cLBP. Some studies indicate a link between muscle degeneration and cLBP, suggesting their fundamental role in the development and persistence of cLBP [8, 10–14], while other studies do not [15, 16].

Although there is disagreement about the relationship between paraspinal myodegeneration and cLBP, numerous studies have focused on examining the relationship between paraspinal myodegeneration and the duration and intensity of cLBP. Common to all studies is that they predominantly investigated solely the influence of the paraspinal muscle cross-sectional area (CSA) [17–19]. However, changes in functional cross-sectional area (FCSA) and fat infiltration (FI) are equally significant in understanding their impact on cLBP, as fat can replace muscle, resulting in CSA remaining constant while FI increases. Uçar et al [13] showed that the rate of abdominal adipose tissue was a significant and independent factor in the severity of low back pain and suggested that increased FI is a possible determinant influencing pain intensity. Furthermore, research indicates a close correlation between cLBP and individuals’ quality of life [20, 21]. However, whether the decrease in quality of life in cLBP patients is associated with the quality of lumbar paraspinal muscles remains unknown.

Therefore, this study aimed to investigate the relationship between the quality of paraspinal muscles and pain intensity, pain duration, and quality of life in patients with cLBP. It was hypothesized that there is (i) a negative correlation between CSA and FCSA, as well as (ii) a positive correlation between FI and pain intensity and duration. However, (iii) the expected correlation regarding quality-of-life reverses.

## Materials and methods

### Participant selection

The study received approval from the local ethics committee (approval number: EA1/058/21), and all participants consented to the use of their data in this research. The data was gathered through a population-based retrospective study as part of an ongoing large cohort study. The study, reported in accordance with the STROBE guidelines, collected lumbar magnetic resonance imaging (MRI) scans between January 2022 and December 2023. These scans comprised 119 individuals with no history of back pain (57 males, 62 females), aged 19–64, and 136 cLBP patients (66 males, 70 females), aged 20–63. The no-back pain (no-BP) group consists of individuals who have never experienced pain in their entire back or pelvis and have not undergone surgery on the spine, pelvis, and hip joints. The cLBP group comprises patients with persistent LBP lasting more than 12 weeks. Patients with prior vertebral fractures, radiculopathies causing muscle weakness, previous spinal surgery, or non-spinal conditions that significantly reduce daily activity (such as cardiovascular diseases, heart failure, myocardial ischemia, neurological disorders, or malignancies) were excluded from this study.

### Participants’ characteristics

Information on the patient’s age, sex, body height, and weight, as well as their pain status (pain intensity via VAS [22] and pain duration) and quality of life (36-item Short-Form Health Survey, SF-36 [23]) were assessed via questionnaires and physical measurements. The final pain intensity value is based on the average pain intensity over the past 12 weeks. The SF-36 questionnaire consists of eight scaled scores, covering physical (including physical functioning, role—physical, bodily pain, and general health), and mental health (including validity, social function, role-emotional, and mental health). These scores are calculated by adding the weighted responses in their respective sections and then converted to a 0–100 scale. The lower the score, the greater the disability.

### MRI—muscle segmentation

In a pilot study, we explored various methods and MRI protocols to achieve optimal distinction among the different muscles in the lumbar spine [24]. The imaging procedures were carried out using a Siemens Lumina 3.0-T MRI system (Siemens AG). We employed T2-weighted turbo spin echo sequences to obtain both axial and sagittal images. The specific parameters for the axial T2 images were set with a repetition time of 4.000, an echo time of 113, and a slice thickness of 3 mm.

**Fig. 1:**
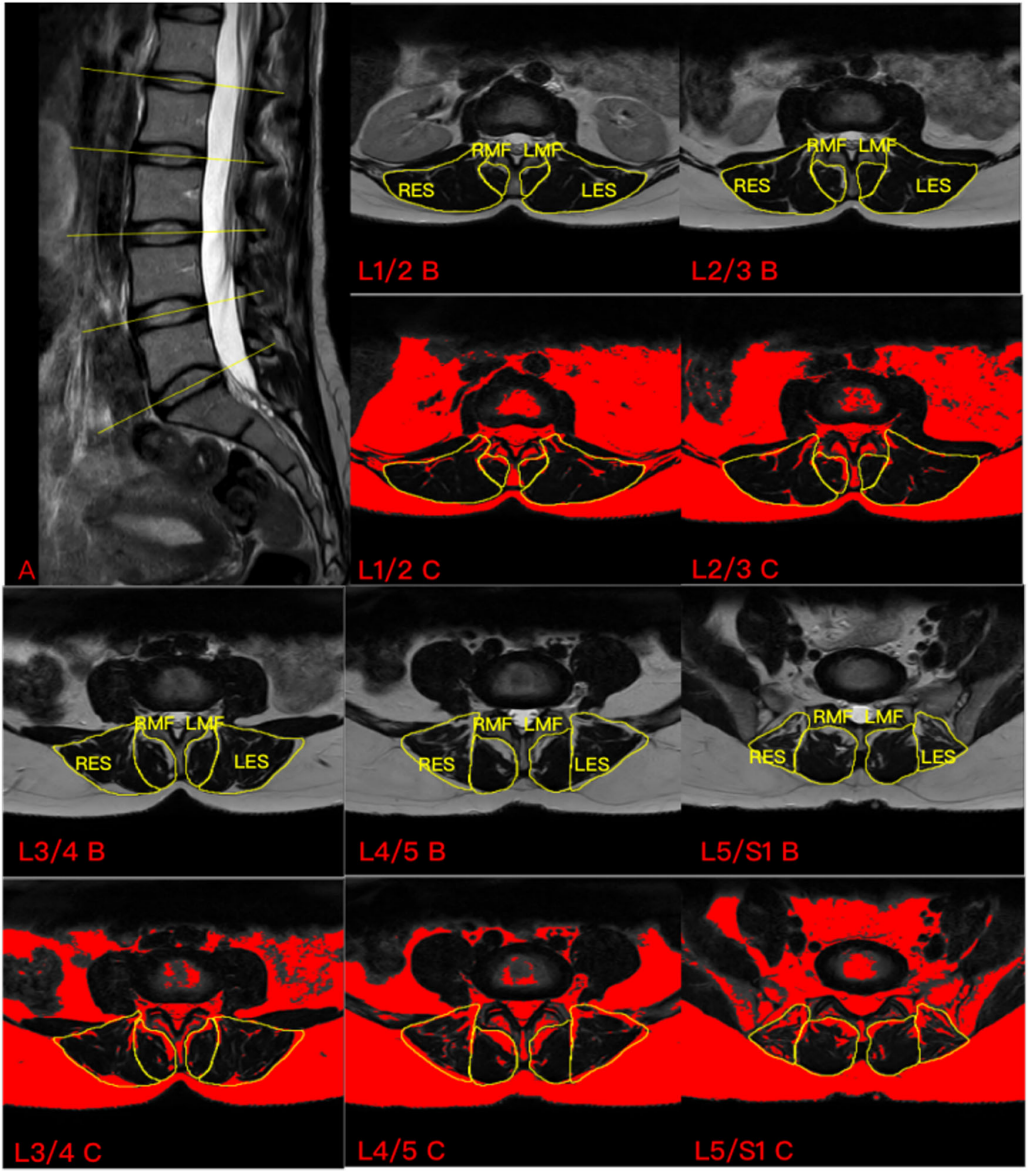
MRI of a subject’s lumbar spine and target muscle measurements. **A** Sagittal view of the lumbar spine, the yellow line represents the location of the measured cross-sectional MRI. **B** (L1/2) CSA of the MF and ES at the L1/2 segment. **C** (L1/L2) FCSA of the MF and ES at the L1/2 segment. LMF, left multifidus; LES, left erector spinae; RMF, right multifidus; RES, right erector spinae

The muscles of interest, that is the CSA of MF and ES (Fig. 1), were examined. After the region of interest (i.e., the target muscle) was delineated using ImageJ (version 1.53, National Institutes of Health), the thresholding algorithm proposed by Otsu was used to differentiate between muscle fibers and fatty infiltration [25]. Fat area and FCSA (the pure muscle area excluding fat) were obtained using this algorithm. All measurements were first conducted twice by an orthopedic resident who received specialized training in MRI muscle assessment. Subsequently, a radiologist performed an additional measurement on the same scans. The average of the three measurements was then used for data analysis. The order of measurements was randomized, with a time interval of at least one month between the two consecutive measurements.

### MRI parameters

Muscle CSA, functional cross-section area (FCSA), and fat infiltration percentage (FI), were calculated for MF and ES. FI is expressed as a percentage: FI = (Fat area/CSA) × 100. Measurements were taken three times at the mid-disc level of all lumbar segments (L1/2–L5/S1) for the CSA, FCSA, and fat area of both the MF and ES muscles. Segmentation sections for each intervertebral level were precisely delineated between each MRI pediculo-transverse slice.

### Statistics

Descriptive statistics were employed to compute baseline information for demographic and clinical variables. For radiographic measurements, the inter- and intra-rater reliability was assessed using intraclass correlation coefficients. Binary logistic regression was employed to explore the distinctions in lumbar paraspinal muscle characteristics between the no-BP group and the cLBP group. Multiple linear regression was utilized to investigate the relationship between degenerative changes in paraspinal muscles and cLBP (both its duration and intensity), as well as quality of life. In Model 1, no confounding factors were adjusted. In Model 2, adjustments were made for sex, age, and BMI. Based on Cohen’s [26] recommendations, effect size (partial eta squared) conventions were employed, categorizing effect sizes as “small” (0.01), “medium” (0.06), and “large” (0.14). Statistical analysis was performed using Statistical Package for the Social Sciences version 23.0 (SPSS Inc). Statistical significance was defined as *p* < 0.05.

## Results

Out of the 255 patients included in this study, demographic data are presented in Table 1. Subsequent sections will exclusively display the results pertaining to the left side. The results on the right are similar and thus provided in Supplementary Tables 1–4. According to Portney and Watkins’s study [27], the intra- and inter-rater reliability exceeded 0.75, which indicates excellent consistency.

**Table 1:** Participant demographics

	no-BP	cLBP
Sex, (females:males)	62:57	70:66
Mean ± SD		
Age	40.82 ± 12.70	41.43 ± 10.54
BMI	23.07 ± 2.31	23.70 ± 3.08
Intensity of pain	–	4.00 ± 1.47
Duration of pain, (years)	–	9.81 ± 8.61
SF36 scores	–	530.55 ± 124.41

SF36 scores are calculated by summing the weighted responses within each section and then scaling the total to a 0–100 range

### Differences between no-BP and cLBP group

Significant differences between no-BP subjects and cLBP patients were only found in the lower lumbar spine. Compared to the no-BP group, the cLBP patients showed larger CSA of the ES at L5/S1 (OR: 1.086, 95% CI: 1.018–1.158, *p* = 0.01). Conversely, the cLBP group exhibited smaller FCSA of the ES at the L4/L5 level (OR: 0.890, 95% CI: 0.824–0.961, *p* < 0.01) compared to the no-BP group. Furthermore, the ES and MF FI at the L4/L5 and L5/S1 levels demonstrated lower values in the no-BP group than in the cLBP group (*p* < 0.01). No other statistically significant differences were detected (Table 2).

**Table 2:** Correlation between muscle CSA, FCSA, and FI and cLBP

		Mean, (SD)		OR (95% CI)	*p*-value
		no-BP	cLBP		
CSA, (cm^2^)					
L1/L2	MF	2.44 (0.67)	2.49 (0.67)	1.105 (0.722–1.691)	0.65
	ES	18.55 (4.68)	19.04 (4.28)	1.017 (0.935–1.106)	0.69
L2/L3	MF	3.93 (1.01)	4.03 (1.01)	1.109 (0.836–1.471)	0.47
	ES	18.88 (4.19)	19.54 (4.02)	1.036 (0.948–1.133)	0.43
L3/L4	MF	6.23 (1.39)	6.44 (1.37)	1.120 (0.910–1.377)	0.28
	ES	18.14 (3.88)	18.43 (3.81)	0.995 (0.913–1.085)	0.91
L4/L5	MF	9.70 (1.85)	9.95 (1.82)	1.086 (0.921–1.238)	0.39
	ES	16.66 (3.81)	16.58 (3.57)	0.975 (0.905–1.050)	0.50
L5/S1	MF	11.67 (1.96)	12.10 (2.02)	1.112 (0.973–1.272)	0.12
	ES	**10.93 (4.25)**	**12.35 (4.08)**	**1.086 (1.018**–**1.158)**	**0.01**
FCSA, (cm^2^)					
L1/L2	MF	2.27 (0.65)	2.34 (0.66)	1.210 (0.766–1.913)	0.41
	ES	17.73 (4.54)	18.11 (4.22)	1.010 (0.925–1.103)	0.82
L2/L3	MF	3.58 (0.98)	3.70 (0.99)	1.147 (0.846–1.556)	0.38
	ES	17.88 (4.18)	18.34 (4.03)	1.019 (0.932–1.113)	0.68
L3/L4	MF	5.52 (1.34)	5.70 (1.35)	1.128 (0.897–1.418)	0.30
	ES	16.61 (3.90)	16.66 (3.83)	0.976 (0.895–1.065)	0.59
L4/L5	MF	8.47 (1.88)	8.17 (1.71)	0.854 (0.722–1.010)	0.07
	ES	**14.83 (3.91)**	**13.62 (3.71)**	**0.890 (0.824**–**0.961)**	**<** **0.01**
L5/S1	MF	9.67 (2.01)	9.38 (1.91)	0.892 (0.769–1.035)	0.13
	ES	8.89 (3.86)	9.15 (3.94)	1.014 (0.947–1.085)	0.70
FI, (%)					
L1/L2	MF	6.99 (8.66)	6.28 (8.21)	0.989 (0.957–1.023)	0.52
	ES	4.44 (3.32)	4.98 (3.68)	1.051 (0.964–1.146)	0.26
L2/L3	MF	8.93 (7.42)	8.14 (8.58)	0.987 (0.951–1.023)	0.47
	ES	5.47 (3.75)	6.32 (4.49)	1.062 (0.984–1.146)	0.12
L3/L4	MF	11.47 (7.30)	11.48 (8.69)	0.999 (0.963–1.037)	0.97
	ES	8.68 (5.23)	9.89 (5.96)	1.045 (0.989–1.105)	0.12
L4/L5	MF	**12.90 (7.35)**	**17.64 (9.41)**	**1.114 (1.067**–**1.162)**	**<** **0.01**
	ES	**11.34 (7.42)**	**18.29 (9.28)**	**1.146 (1.095**–**1.200)**	**<** **0.01**
L5/S1	MF	**17.43 (8.29)**	**22.54 (8.62)**	**1.121 (1.075**–**1.170)**	**<** **0.01**
	ES	**19.34 (11.00)**	**27.79 (12.98)**	**1.082 (1.051**–**1.113)**	**<** **0.01**

Adjusted for age, sex and BMI

*CSA* cross-sectional area, *FCSA* functional cross-sectional area, *FI* fat infiltration, *MF* multifidus, *ES* erector spinae, *OR* odds ratio, *95% CI* 95% confidence interval

Bold values mean *p* < 0.05

### Effect of pain intensity

Apart from the CSA (*p* = 0.02, beta = 0.363, partial η^2^ = 0.04) and FCSA (*p* = 0.03, beta = 0.341, partial η^2^ = 0.03) of the L2/3 MF muscle, no significant association was found between the CSA, FCSA, and FI of paraspinal muscles and pain intensity, even before or after adjusting for age, sex, and BMI (Supplementary Tables 5–8). The effect sizes observed were small (partial η^2^ ≤ 0.03).

### Effect of pain duration

Prior to adjusting for age, sex and BMI, the FI of paraspinal muscles showed a positive correlation with the duration of cLBP across all lumbar spinal segments. After adjusting for relevant confounders, positive correlations could still be observed in the L2/3, L3/4, and L4/5 segments (*p* < 0.05) (Table 3). The beta coefficient ranged from 0.178 to 0.421, with a partial η^2^ range of 0.03 to 0.06. Before adjusting for confounding factors, the CSA and FCSA of paraspinal muscles in most lumbar vertebral segments showed an inverse relationship with the duration of cLBP (Supplementary Tables 9 and 10). However, these correlations ceased to be significant after controlling for age, sex, and BMI, except for the CSA of the L1/2 MF muscle (*p* = 0.02, beta = 2.871, partial η^2^ = 0.04). The beta coefficient ranged from − 0.746 to 2.871, with a partial η^2^ of less than 0.04 (Supplementary Table 11).

**Table 3:** Correlation between muscle FI (%) and duration of cLBP

		Model 1		Model 2	
		95% CI	*p*-value	95% CI	*p*-value
L1/L2	MF	**0.307 (0.136–0.479)**	**<** **0.01**	0.126 (− 0.062 to 0.314)	0.19
	ES	**0.738 (0.359–1.117)**	**<** **0.01**	0.366 (− 0.069 to 0.800)	0.10
L2/L3	MF	**0.359 (0.199–0.519)**	**<** **0.01**	**0.204 (0.017–0.392)**	**0.03**
	ES	**0.681 (0.375–0.987)**	**<** **0.01**	**0.421 (0.057–0.785)**	**0.02**
L3/L4	MF	**0.412 (0.258–0.566)**	**<** **0.01**	**0.269 (0.089–0.449)**	**<** **0.01**
	ES	**0.560 (0.333–0.788)**	**<** **0.01**	**0.408 (0.124–0.693)**	**<** **0.01**
L4/L5	MF	**0.365 (0.222–0.509)**	**<** **0.01**	**0.210 (0.039–0.381)**	**0.02**
	ES	**0.293 (0.146–0.440)**	**<** **0.01**	**0.178 (0.003–0.353)**	**0.046**
L5/S1	MF	**0.314 (0.152–0.476)**	**<** **0.01**	0.113 (− 0.078 to 0.303)	0.24
	ES	**0.130 (0.018–0.241)**	**0.02**	0.049 (− 0.065 to 0.164)	0.39

Model 1 unadjusted

Model 2 adjusted for age, sex, and BMI

*FI* fat infiltration, *MF* multifidus, *ES* erector spinae, *95% CI* 95% confidence interval

Bold values mean *p* < 0.05

### Effect of health-related quality of life

A similar positive correlation between the CSA of the MF muscle at L3/4, L4/5, and L5/S1 segments and SF36 scores were observed, both before and after adjusting for age, sex, and BMI (Table 4). Moreover, the FCSA of the MF muscle at L4/5 and L5/S1 segments also exhibits a positive correlation with SF36 scores. No correlation was observed between the CSA and FCSA of the ES muscles (partial η^2^ < 0.01, *p* > 0.05, beta: − 3.967 to 2.333; Supplementary Tables 12–14). Moreover, neither the FI of MF nor ES showed any correlation with the SF36 scores (partial η^2^ ≤ 0.01, *p* > 0.05, beta: − 0.758 to 1.968).

**Table 4:** Correlation between MF muscle CSA (cm^2^) and FCSA (cm^2^) and SF-36

		Model 1		Model 2	
		95% CI	*p*	95% CI	*p*
CSA	L1/L2	8.646 (− 24.155 to 41.446)	0.60	12.561 (−26.803 to 51.926)	0.53
	L2/L3	− 6.135 (− 27.182 to 14.912)	0.57	− 8.984 (− 34.193 to 16.226)	0.48
	L3/L4	13.229 (− 2.103 to 28.561)	0.09	**19.864 (2.187**–**37.541)**	**0.03**
	L4/L5	**12.012 (0.521**–**23.503)**	**0.04**	**15.320 (2.962**–**27.678)**	**0.02**
	L5/S1	**14.584 (4.344**–**24.825)**	**<** **0.01**	**16.926 (6.222**–**27.630)**	**<** **0.01**
FCSA	L1/L2	− 0.747 (− 31.584 to 33.078)	0.96	0.935 (− 40.219 to 42.090)	0.96
	L2/L3	− 8.561 (− 29.987 to 12.865)	0.43	− 14.065 (− 41.078 to 12.947)	0.31
	L3/L4	8.888 (− 6.834 to 24.610)	0.27	14.635 (− 4.818 to 34.089)	0.14
	L4/L5	10.778 (− 1.539 to 23.094)	0.09	**16.173 (1.283–31.063)**	**0.03**
	L5/S1	**12.259 (1.697–22.821)**	**0.02**	**16.354 (4.089–28.620)**	**<** **0.01**

Model 1 unadjusted

Model 2 adjusted for age, sex, and BMI

*CSA* cross-sectional area, *FCSA* functional cross-sectional area, *MF* multifidus, *ES* erector spinae, *95% CI* 95% confidence interval

Bold values mean *p* < 0.05

## Discussion

In the present study, 255 patients with and without cLBP were investigated to explore the relationship between markers of muscle composition and cLBP. Significant differences were observed in paraspinal muscle characteristics, particularly in the lower lumbar spine. cLBP patients exhibited a larger CSA of the ES at the L5/S1 level but a smaller FCSA of the ES at the L4/L5 level compared to the no-BP group. This association may suggest a compensatory mechanism in cLBP patients, where the body increases muscle size to alleviate pain duration and intensity. However, whether muscle enlargement fully offsets pain impact or reflects a natural response to provide additional spinal support remains uncertain.

Additionally, higher FI of both ES and MF at the L4/L5 and L5/S1 levels in the cLBP group indicates muscle degeneration or pathological changes associated with cLBP conditions. This discovery aligns with prior research and underscores that higher FI may compromise muscle functionality, leading to diminished muscular quality. A meta-analysis found a significant increase in FI in paraspinal muscles in individuals with LBP [10]. Findings of Goubert et al [28] further indicate that, compared to recurrent LBP, there is a higher level of FI in the MF and ES muscles in cases of cLBP.

While consistent with existing literature linking higher FI levels to increased cLBP risk, our study did not identify a significant correlation between FI and cLBP intensity. Instead, our results suggest that factors beyond muscle composition, such as pain processing mechanisms and psychosocial factors [29, 30], may play a more substantial role in determining cLBP intensity. Additionally, there’s a possibility that aspects of the pain experience, such as the fear of pain and individual beliefs about pain, which pain scales might not fully capture, could be linked to muscle morphology [31–33]. These factors might influence the perception and experience of pain chronically, contributing to its impact on individuals, even if the pain intensity measured by conventional scales does not reflect this effect. Overall, the impact of pain levels may be influenced by various factors, necessitating further research to better understand their role in the development of cLBP.

A significant finding is the positive correlation between FI and cLBP duration, implying a progressive relationship between FI and cLBP over time. This highlights the dynamic nature of muscle degeneration in cLBP and suggests that FI may worsen with prolonged pain. These findings underscore the potential significance of FI in cLBP pathophysiology and its clinical implications for disease management. Cooley et al [34] have confirmed that the quality of the MF muscle in patients affects the duration of LBP, but it is unrelated to the intensity of the LBP. Strategies targeting FI reduction within paraspinal muscles, such as focused exercise programs or pharmacological interventions, may hold promise in mitigating cLBP progression or severity. Matthew et al [35] demonstrated that graded sensorimotor retraining, in comparison to a sham procedure and attention control, resulted in a significant reduction in pain intensity at the 18-week mark. Nonetheless, additional research is warranted to clarify the underlying mechanisms driving the FI-cLBP association and evaluate intervention efficacy.

At lumbar levels L3/4, L4/5, and L5/S1, more extensive and stronger MF muscles positively correlated with physical and psychological health-related quality of life, as assessed by the SF-36 questionnaire. These findings underscore the importance of lumbar muscle health in maintaining spinal stability and overall well-being. Hlaing et al [36], in turn, demonstrated that core stability training not only augments the thickness of the MF muscles but also effectively mitigates LBP. Furthermore, it enhances patients’ proprioception and equilibrium, reduces functional limitations, and diminishes apprehension toward physical activity.

Multilevel studies have their benefits, Julio et al [37] demonstrate that individual lumbar levels cannot represent the entire lumbar spine, and multilevel studies can better understand degenerative changes in paraspinal muscle characteristics across the lumbar spine. Therefore, we observed a positive correlation between the CSA of the MF muscle and the duration and intensity of cLBP at certain stages. This may indicate compensatory muscle performance during the prolonged course of symptoms in patients with cLBP. However, it is important to note that CSA, as a parameter, has its limitations, as it fails to exclude the influence of fat content. Additionally, this study conducted numerous tests, which may have led to type I errors. Thus, additional research is required to validate the correlation between CSA and the duration and intensity of cLBP.

The current study has several limitations that should be considered when interpreting the findings. Firstly, only 2.9% of the total cLBP patients experienced severe pain (VAS score ≥ 7), potentially limiting the generalizability of our findings to patients with severe cLBP. Additional research is needed to investigate the association between severe cLBP and changes in paraspinal muscle characteristics. Moreover, other relevant confounding factors may not have been included in our study or were not present in the available data, such as genetic predispositions. This underscores the need for larger-scale studies to validate our results and address these limitations effectively.

## Conclusion

This study underscores the complexity of the relationship between lumbar paraspinal muscle degeneration and cLBP. In cLBP patients, the paraspinal muscle FI was positively correlated with cLBP and duration of pain but not with pain intensity. Additionally, the CSA and FCSA of MF muscle were positively correlated with the quality of life in cLBP patients. Studying the paraspinal muscles across multiple segments can help comprehend the association between muscle degeneration and cLBP. Further study is imperative to clarify the underlying mechanisms and clinical significance of these findings, thus paving the way for tailored interventions to relieve the back pain burden experienced by affected individuals.

## Supplementary information

ELECTRONIC SUPPLEMENTARY MATERIAL

## Abbreviations

cLBP: Chronic low back pain
CSA: Cross-sectional area
ES: Erector spinae
FCSA: Functional cross-sectional area
FI: Fat infiltration
MF: Multifidus
MRI: Magnetic resonance imaging
no-BP: No-back pain
