# Supplementary material for: Quality of lumbar paraspinal muscles in patients with chronic low back pain and its relationship to pain duration, pain intensity, and quality of life
Source: Eur Radiol. 2024 Dec 7;35(6):3652–60. doi: 10.1007/s00330-024-11236-y (PMC12081590; doi:10.1007/s00330-024-11236-y)
Supplement: Supplementary file 1 — ELECTRONIC SUPPLEMENTARY MATERIAL [file 330_2024_11236_MOESM1_ESM.pdf]

Quality of Lumbar Paraspinal Muscles in Patients with Chronic Low Back Pain and its Relationship to Pain Duration, Pain Intensity, and Quality of Life

ELECTRONIC SUPPLEMENTARY MATERIAL

Supplemental Table 1. Correlation between muscle CSA, FCSA and FI and cLBP on the right side.

Supplemental Table 2. Correlation between muscle CSA, FCSA and FI and intensity of cLBP on the right side.

Supplemental Table 3. Correlation between muscle CSA, FCSA and FI and duration of LBP on the right side.

Supplemental Table 4. Correlation between muscle CSA, FCSA and FI and SF36 on the right side.

Supplemental Table 5. Correlation between muscle CSA and intensity of cLBP on the left side.

Supplemental Table 6. Correlation between muscle FCSA and intensity of cLBP on the left side.

Supplemental Table 7. Correlation between muscle FI and intensity of cLBP on the left side.

Supplemental Table 8. Effect size between muscle CSA, FCSA, FI and intensity of cLBP.

Supplemental Table 9. Correlation between muscle CSA and duration of cLBP on the left side.

Supplemental Table 10. Correlation between muscle FCSA and duration of cLBP on the left side.

Supplemental Table 11. Effect size between muscle CSA, FCSA, FI and duration of cLBP.

Supplemental Table 12. Correlation between ES muscle CSA and FCSA and SF-36 on the left side.

Supplemental Table 13. Correlation between muscle FI and SF-36 on the left side.

Supplemental Table 14. Effect size between muscle CSA, FCSA, FI and SF36.

Eur Radiol (2024) Liu S, Reitmaier S, Mödl L, et al.

Supplemental Table 1. Correlation between muscle CSA, FCSA and FI and cLBP on the right side.

		Mean (SD)		OR (95%CI)	p-value
		no-BP	cLBP		
CSA (cm^2)					
L1/L2	MF	2.56 (0.66)	2.65 (0.64)	1.249 (0.804-1.941)	0.32
	ES	18.35 (4.45)	18.88 (4.33)	1.021 (0.935-1.115)	0.65
L2/L3	MF	4.10 (1.02)	4.10 (1.01)	0.959 (0.728-1.265)	0.77
	ES	18.66 (4.38)	19.29 (3.87)	1.036 (0.949-1.132)	0.42
L3/L4	MF	6.63 (1.41)	6.92 (1.39)	1.178 (0.960-1.444)	0.12
	ES	17.96 (3.85)	18.43 (3.85)	1.017 (0.932-1.110)	0.70
L4/L5	MF	**9.71 (1.77)**	**10.21 (1.72)**	**1.191 (1.014-1.398)**	**0.03**
	ES	16.27 (3.61)	15.90 (3.27)	0.938 (0.862-1.020)	0.13
L5/S1	MF	**11.77 (1.90)**	**12.51 (1.95)**	**1.233 (1.068-1.425)**	**<0.01**
	ES	**10.75 (3.64)**	**11.98 (3.68)**	**1.094 (1.017-1.178)**	**0.02**
FCSA (cm^2)					
L1/L2	MF	2.39 (0.66)	2.49 (0.65)	1.334 (0.837-2.125)	0.23
	ES	17.52 (4.32)	17.71 (4.26)	0.980 (0.894-1.075)	0.68
L2/L3	MF	3.70 (0.99)	3.71 (1.00)	0.960 (0.707-1.303)	0.79
	ES	17.68 (4.30)	17.82 (3.95)	0.980 (0.897-1.071)	0.66
L3/L4	MF	5.84 (1.35)	6.02 (1.31)	1.135 (0.896-1.436)	0.29
	ES	16.57 (3.81)	16.44 (3.88)	0.953 (0.871-1.043)	0.30
L4/L5	MF	8.44 (1.81)	8.19 (1.63)	0.852 (0.710-1.022)	0.09
	ES	**14.61 (3.70)**	**12.86 (3.44)**	**0.811 (0.740-0.888)**	**<0.01**
L5/S1	MF	9.88 (1.98)	9.56 (2.04)	0.875 (0.752-1.017)	0.08
	ES	8.96 (3.49)	8.76 (3.58)	0.971 (0.900-1.047)	0.45
FI (%)					

L1/L2	MF	6.72 (7.09)	6.06 (7.34)	0.985 (0.948-1.024)	0.46
	ES	**4.52 (3.33)**	**6.31 (4.15)**	**1.183 (1.085-1.290)**	**<0.01**
L2/L3	MF	9.68 (8.29)	9.42 (9.32)	0.996 (0.964-1.030)	0.82
	ES	**5.40 (3.20)**	**7.85 (4.99)**	**1.241 (1.133-1.359)**	**<0.01**
L3/L4	MF	11.97 (7.51)	12.88 (9.20)	1.020 (0.983-1.057)	0.30
	ES	**7.96 (4.85)**	**11.10 (6.16)**	**1.160 (1.088-1.237)**	**<0.01**
L4/L5	MF	**13.24 (7.58)**	**19.53 (10.11)**	**1.137 (1.089-1.187)**	**<0.01**
	ES	**10.57 (7.07)**	**19.60 (9.79)**	**1.207 (1.146-1.271)**	**<0.01**
L5/S1	MF	**16.30 (8.02)**	**23.69 (9.63)**	**1.158 (1.109-1.210)**	**<0.01**
	ES	**17.55 (10.28)**	**28.50 (12.53)**	**1.130 (1.091-1.171)**	**<0.01**

CSA= cross-sectional area; FCSA= functional cross-sectional area; FI= fat infiltration; MF= multifidus; ES= erector spinae; OR= odds ratio; 95%CI = 95% Confidence Interval

Adjusted for age, sex and BMI

Supplemental Table 2. Correlation between muscle CSA (cm^2), FCSA (cm^2) and FI (%) and intensity of cLBP on the right side.

		Model 1		Model 2		Model 3	
		Beta coefficient (95% CI)	P	Beta coefficient (95% CI)	P	Beta coefficient (95% CI)	P
L1/L2	MF	0.237 (-0.226- 0.699)	0.31	0.250 (-0.237-0.736)	0.31	-0.001 (-0.040-0.038)	0.97
	ES	-0.047 (-0.138-0.044)	0.31	-0.026 (-0.123-0.071)	0.60	-0.060 (-0.132-0.012)	0.10
L2/L3	MF	0.162 (-0.121-0.445)	0.26	0.210 (-0.101-0.522)	0.18	-0.003 (-0.035-0.029)	0.84
	ES	-0.074 (-0.163-0.016)	0.11	-0.050 (-0.140-0.039)	0.27	-0.035 (-0.099-0.029)	0.28
L3/L4	MF	-0.043 (-0.244-0.157)	0.67	-0.025 (-0.258-0.208)	0.83	<0.001 (-0.034-0.034)	0.99
	ES	-0.005 (-0.092-0.082)	0.91	0.015 (-0.074-0.103)	0.74	-0.029 (-0.083-0.025)	0.29
L4/L5	MF	-0.052 (-0.211-0.107)	0.52	-0.016 (-0.206-0.174)	0.87	-0.009 (-0.040-0.021)	0.54
	ES	-0.042 (-0.127-0.044)	0.34	0.001 (-0.086-0.088)	0.98	-0.027 (-0.060-0.005)	0.10
L5/S1	MF	-0.050 (-0.186-0.086)	0.47	-0.014 (-0.162-0.135)	0.86	-0.011 (-0.043-0.021)	0.50
	ES	-0.006 (-0.078-0.066)	0.87	0.002 (-0.077-0.080)	0.97	-0.010 (-0.032-0.013)	0.40

CSA= cross-sectional area; FCSA= functional cross-sectional area; FI= fat infiltration; MF= multifidus; ES= erector spinae; 95%CI= 95% Confidence Interval

Adjusted for age, sex and BMI

Model 1 correlation between muscle CSA and intensity of cLBP

Model 2 correlation between muscle FCSA and intensity of cLBP

Model 3 correlation between muscle FI and intensity of cLBP

Supplemental Table 3. Correlation between muscle CSA (cm^2), FCSA (cm^2) and FI (%) and duration of LBP on the right side.

		Model 1		Model 2		Model 3	
		Beta coefficient (95% CI)	P	Beta coefficient (95% CI)	P	Beta coefficient (95% CI)	P
L1/L2	MF	1.655 (-0.805-4.115)	0.19	1.040 (-1.558-3.637)	0.43	0.178 (-0.027-0.382)	0.09
	ES	-0.088 (-0.575-0.399)	0.72	-0.251 (-0.765-0.262)	0.34	**0.527 (0.149-0.905)**	**<0.01**
L2/L3	MF	0.499 (-1.016-2.015)	0.52	-0.375 (-2.047-1.296)	0.66	**0.230 (0.063-0.397)**	**<0.01**
	ES	-0.045 (-0.525-0.435)	0.85	-0.181 (-0.657-0.296)	0.46	**0.436 (0.101-0.771)**	**0.01**
L3/L4	MF	0.430 (-0.639-1.499)	0.43	-0.427 (-1.668-0.813)	0.50	**0.254 (0.080-0.429)**	**<0.01**
	ES	-0.075 (-0.539-0.390)	0.75	-0.179 (-0.650-0.292)	0.45	**0.277 (-0.007-0.560)**	**0.056**
L4/L5	MF	-0.026 (-0.873-0.821)	0.95	-0.744 (-1.749-0.262)	0.15	**0.187 (0.029-0.345)**	**0.02**
	ES	-0.143 (-0.600-0.313)	0.54	-0.147 (-0.612-0.318)	0.53	0.041 (-0.133-0.215)	0.64
L5/S1	MF	-0.363 (-1.089-0.362)	0.32	**-0.781 (-1.560- -0.001)**	**0.050**	0.159 (-0.008-0.326)	0.06
	ES	-0.041 (-0.426-0.344)	0.83	-0.156 (-0.574-0.263)	0.46	0.058 (-0.063-0.179)	0.34

CSA= cross-sectional area; FCSA= functional cross-sectional area; FI= fat infiltration; MF= multifidus; ES= erector spinae; 95%CI= 95% Confidence Interval

Adjusted for age, sex and BMI

Model 1 correlation between muscle CSA and duration of cLBP

Model 2 correlation between muscle FCSA and duration of cLBP

Model 3 correlation between muscle FI and duration of cLBP

Supplemental Table 4. Correlation between muscle CSA (cm^2), FCSA (cm^2) and FI (%) and SF36 on the right side.

		Model 1		Model 2		Model 3	
		Beta coefficient (95% CI)	P	Beta coefficient (95% CI)	P	Beta coefficient (95% CI)	P
L1/L2	MF	-1.478 (-40.990-38.033)	0.94	0.112 (-41.431-41.654)	0.996	-0.212 (-3.517-3.094)	0.90
	ES	6.994 (-0.682-14.670)	0.07	7.327 (-0.800-15.455)	0.08	0.241 (-5.966-6.447)	0.94
L2/L3	MF	-9.489 (-33.658-14.679)	0.44	-14.646 (-41.214-11.923)	0.28	0.527 (-2.212-3.266)	0.70
	ES	**9.105 (1.610-16.600)**	**0.02**	**8.972 (1.505-16.438)**	**0.02**	0.630 (-6.102-4.482)	0.82
L3/L4	MF	**17.582 (0.760-34.404)**	**0.04**	14.720 (-4.944-34.383)	0.14	1.415 (-1.445-4.275)	0.33
	ES	1.308 (-6.107-8.724)	0.73	0.817 (-6.713-8.347)	0.83	1.035 (-3.544-5.615)	0.66
L4/L5	MF	**14.522 (1.249-27.795)**	**0.03**	11.797 (-4.249-27.842)	0.15	1.135 (-1.431-3.701)	0.38
	ES	0.594 (-6.701-7.889)	0.87	-0.638 (-8.071-6.795)	0.87	1.121 (-1.650-3.892)	0.43
L5/S1	MF	**19.218 (8.082-30.354)**	**<0.01**	13.848 (1.461-26.235)	0.03	1.117 (-1.574-3.809)	0.41
	ES	2.189 (-3.942-8.319)	0.48	1.480 (-5.211-8.171)	0.66	0.452 (-1.487-2.391)	0.65

CSA= cross-sectional area; FCSA= functional cross-sectional area; FI= fat infiltration; MF= multifidus; ES= erector spinae; 95%CI= 95% Confidence Interval

Adjusted for age, sex and BMI

Model 1 correlation between muscle CSA and SF36

Model 2 correlation between muscle FCSA and SF36

Model 3 correlation between muscle FI and SF36

Supplemental Table 5. Correlation between muscle CSA (cm^2) and intensity of cLBP on the left side.

		model 1		model 2	
		95%CI	P	95%CI	P
L1/L2	MF	-0.044 (-0.431-0.343)	0.82	0.120 (-0.343- 0.583)	0.61
	ES	-0.033 (-0.091-0.025)	0.27	-0.027 (-0.115-0.061)	0.55
L2/L3	MF	0.158 (-0.088-0.405)	0.21	**0.363 (0.072-0.653)**	**0.02**
	ES	-0.003 (-0.065-0.059)	0.93	0.028 (-0.061-0.117)	0.54
L3/L4	MF	-0.008 (-0.190-0.175)	0.93	0.064 (-0.148-0.275)	0.55
	ES	0.012 (-0.053-0.078)	0.72	0.046 (-0.041-0.133)	0.30
L4/L5	MF	-0.059 (-0.196-0.078)	0.40	-0.035 (-0.183-0.114)	0.65
	ES	-0.021 (-0.091-0.049)	0.56	-0.018 (-0.095-0.059)	0.64
L5/S1	MF	-0.049 (-0.173-0.074)	0.43	-0.041 (-0.171-0.089)	0.53
	ES	0.050 (-0.010-0.111)	0.10	0.049 (-0.016-0.114)	0.14

CSA= cross-sectional area; MF= multifidus; ES= erector spinae; 95%CI = 95% Confidence Interval

Model 1 without adjusted

Model 2 adjusted for age, sex and BMI

Supplemental Table 6. Correlation between muscle FCSA (cm^2) and intensity of cLBP on the left side.

		model 1		model 2	
		95%CI	P	95%CI	P
L1/L2	MF	-0.063 (-0.444-0.317)	0.74	0.075 (-0.408-0.559)	0.76
	ES	-0.022(-0.081-0.037)	0.46	-0.005(-0.097-0.087)	0.92
L2/L3	MF	0.132 (-0.120-0.384)	0.30	**0.341 (0.028-0.654)**	**0.03**
	ES	0.009 (-0.053-0.071)	0.78	0.049 (-0.039-0.137)	0.27
L3/L4	MF	-0.016 (-0.202-0.170)	0.87	-0.059 (-0.171-0.290)	0.61
	ES	0.021 (-0.044-0.086)	0.53	0.064 (-0.023-0.152)	0.15
L4/L5	MF	-0.047 (-0.193-0.100)	0.53	-0.017 (-0.195-0.161)	0.85
	ES	0.001 (-0.066-0.069)	0.98	0.006 (-0.072-0.084)	0.88
L5/S1	MF	-0.037 (-0.164-0.090)	0.57	-0.033 (-0.181-0.115)	0.66
	ES	0.055 (-0.008-0.117)	0.09	0.056 (-0.014-0.126)	0.12

FCSA= functional cross-sectional area; ES= erector spinae; 95%CI = 95% Confidence Interval

Model 1 without adjusted

Model 2 adjusted for age, sex and BMI

Supplemental Table 7. Correlation between muscle FI (%) and intensity of cLBP on the left side.

		model 1		model 2	
		95%CI	P	95%CI	P
L1/L2	MF	0.009 (-0.022-0.039)	0.58	0.014 (-0.021-0.050)	0.42
	ES	-0.056 (-0.123-0.011)	0.10	-0.077 (-0.158-0.004)	0.06
L2/L3	MF	0.007 (-0.023-0.036)	0.66	0.015 (-0.021-0.050)	0.42
	ES	-0.037 (-0.093-0.018)	0.19	-0.057 (-0.126-0.012)	0.10
L3/L4	MF	0.006 (-0.023-0.035)	0.67	0.008 (-0.027-0.043)	0.65
	ES	-0.019 (-0.061-0.023)	0.38	-0.038 (-0.092-0.017)	0.18
L4/L5	MF	-0.001 (-0.027-0.026)	0.95	-0.005 (-0.038-0.028)	0.76
	ES	-0.015 (-0.041-0.012)	0.28	-0.020 (-0.053-0.013)	0.23
L5/S1	MF	-0.002 (-0.031-0.027)	0.91	-0.004 (-0.040-0.032)	0.81
	ES	-0.013 (-0.033-0.006)	0.17	-0.013 (-0.034-0.008)	0.23

FI= fat infiltration; MF= multifidus; ES= erector spinae; 95%CI = 95% Confidence Interval

Model 1 without adjusted

Model 2 adjusted for age, sex and BMI

Supplemental Table 8. Effect size between muscle CSA, FCSA, FI and intensity of cLBP.

		partial eta squared					
		CSA--Intensity		FCSA--Intensity		FI--Intensity	
		left	right	left	right	left	right
L1/L2	MF	<0.01	<0.01	<0.01	<0.01	<0.01	<0.01
	ES	<0.01	<0.01	<0.01	<0.01	0.03	0.02
L2/L3	MF	0.04	<0.01	0.03	0.01	<0.01	<0.01
	ES	<0.01	0.02	<0.01	<0.01	0.02	<0.01
L3/L4	MF	<0.01	<0.01	<0.01	<0.01	<0.01	<0.01
	ES	<0.01	<0.01	0.02	<0.01	0.01	<0.01
L4/L5	MF	<0.01	<0.01	<0.01	<0.01	<0.01	<0.01
	ES	<0.01	<0.01	<0.01	<0.01	0.01	0.02
L5/S1	MF	<0.01	<0.01	<0.01	<0.01	<0.01	<0.01
	ES	0.02	<0.01	0.02	<0.01	0.01	<0.01

CSA= cross-sectional area; FCSA= functional cross-sectional area; FI= fat infiltration; MF= multifidus; ES= erector spinae

Supplemental Table 9. Correlation between muscle CSA (cm^2) and duration of cLBP on the left side.

		model 1		model 2	
		95%CI	P	95%CI	P
L1/L2	MF	-0.664 (-2.934-1.606)	0.56	**2.871 (0.450-5.292)**	**0.02**
	ES	**-0.531 (-0.862--0.200)**	**<0.01**	0.002 (-0.470-0.474)	0.995
L2/L3	MF	**-1.494 (-2.930--0.058)**	**0.04**	-0.083 (-1.666-1.500)	0.92
	ES	**-0.516 (-0.871--0.160)**	**<0.01**	0.011 (-0.464-0.487)	0.96
L3/L4	MF	-0.832 (-1.896-0.231)	0.12	0.129 (-1.000-1.257)	0.82
	ES	**-0.620 (-0.991--0.249)**	**<0.01**	-0.142 (-0.609-0.325)	0.55
L4/L5	MF	-0.511 (-1.314-0.292)	0.21	-0.002 (-0.794-0.790)	0.995
	ES	**-0.507 (-0.910--0.103)**	**0.01**	-0.126 (-0.536-0.284)	0.54
L5/S1	MF	**-0.734 (-1.453--0.016)**	**0.045**	-0.247 (-0.941-0.447)	0.48
	ES	**-0.399 (-0.753--0.045)**	**0.03**	-0.171 (-0.518-0.177)	0.33

CSA= cross-sectional area; MF= multifidus; ES= erector spinae; 95%CI = 95% Confidence Interval

Model 1 without adjusted

Model 2 adjusted for age, gender, BMI

Supplemental Table 10. Correlation between muscle FCSA (cm^2) and duration of cLBP on the left side.

		model 1		model 2	
		95%CI	P	95%CI	P
L1/L2	MF	-1.990 (-4.202-0.222)	0.08	2.138 (-0.415-4.691)	0.10
	ES	**-0.624 (-0.955--0.292)**	**<0.01**	-0.074 (-0.563-0.415)	0.77
L2/L3	MF	**-2.549 (-3.970--1.128)**	**<0.01**	-0.746 (-2.441-0.949)	0.39
	ES	**-0.637 (-0.985--0.288)**	**<0.01**	-0.104 (-0.576-0.367)	0.66
L3/L4	MF	**-1.921 (-2.964--0.878)**	**<0.01**	-0.679 (-1.903-0.545)	0.28
	ES	**-0.779 (-1.139--0.419)**	**<0.01**	-0.283 (-0.750-0.184)	0.23
L4/L5	MF	**-1.555 (-2.375--0.735)**	**<0.01**	-0.535 (-1.480-0.410)	0.27
	ES	**-0.679 (-1.059--0.300)**	**<0.01**	-0.226 (-0.642-0.189)	0.28
L5/S1	MF	**-1.342 (-2.051--0.633)**	**<0.01**	-0.484 (-1.269-0.300)	0.22
	ES	**-0.485 (-0.849--0.121)**	**<0.01**	-0.190 (-0.566-0.185)	0.32

FCSA= functional cross-sectional area; MF= multifidus; ES= erector spinae; 95%CI =95% Confidence Interval

Model 1 without adjusted

Model 2 adjusted for age, sex and BMI

Supplemental Table 11. Effect size between muscle CSA, FCSA, FI and duration of cLBP.

		partial eta squared					
		CSA--Duration		FCSA--Duration		FI--Duration	
		left	right	left	right	left	right
L1/L2	MF	0.04	0.01	0.02	<0.01	0.01	0.02
	ES	<0.01	<0.01	<0.01	<0.01	0.02	0.05
L2/L3	MF	<0.01	<0.01	<0.01	<0.01	0.03	0.05
	ES	<0.01	<0.01	<0.01	<0.01	0.04	0.05
L3/L4	MF	<0.01	<0.01	<0.01	<0.01	0.06	0.06
	ES	<0.01	<0.01	0.01	<0.01	0.06	0.03
L4/L5	MF	<0.01	<0.01	<0.01	0.02	0.04	0.04
	ES	<0.01	<0.01	<0.01	<0.01	0.03	<0.01
L5/S1	MF	<0.01	<0.01	0.01	0.03	0.01	0.03
	ES	<0.01	<0.01	<0.01	<0.01	<0.01	<0.01

CSA= cross-sectional area; FCSA= functional cross-sectional area; FI= fat infiltration; MF= multifidus; ES= erector spinae

Supplemental Table 12. Correlation between ES muscle CSA (cm^2) and FCSA (cm^2) and SF-36 on the left side.

		model 1		model 2	
		95%CI	P	95%CI	P
CSA	L1/L2	-0.180 (-4.781-5.141)	0.94	2.298 (-5.220-9.817)	0.55
	L2/L3	-3.217 (-8.476-2.041)	0.23	-3.967 (-11.522-3.588)	0.30
	L3/L4	-2.208 (-7.771-3.355)	0.43	-1.777 (-9.230-5.677)	0.64
	L4/L5	-0.816 (-6.776-5.145)	0.79	-0.248 (-6.797-6.302)	0.94
	L5/S1	-0.732 (-5.944-4.479)	0.78	-0.608 (-6.171-4.955)	0.83
FCSA	L1/L2	-0.308 (-4.724-5.340)	0.90	2.333 (-5.463-10.130)	0.56
	L2/L3	-2.830 (-8.080-2.421)	0.29	-3.860 (-11.355-3.635)	0.31
	L3/L4	-1.796 (-7.333-3.741)	0.52	-2.220 (-9.706-5.267)	0.56
	L4/L5	0.381 (-5.347-6.109)	0.90	0.430 (-6.226-7.087)	0.90
	L5/S1	-0.534 (-5.927-4.859)	0.85	-0.504 (-6.516-5.508)	0.87

CSA= cross-sectional area; FCSA= functional cross-sectional area;MF= multifidus; ES= erector spinae; 95%CI = 95% Confidence Interval

Model 1 without adjusted

Model 2 adjusted for age, sex and BMI

Supplemental Table 13. Correlation between muscle FI (%) and SF-36 on the left side.

		model 1		model 2	
		95%CI	P	95%CI	P
L1/L2	MF	1.434 (-1.145-4.013)	0.27	1.953 (-1.044-4.950)	0.20
	ES	-1.186 (-6.951-4.580)	0.69	-0.517 (-7.521-6.487)	0.88
L2/L3	MF	0.848 (-1.624-3.320)	0.50	1.341 (-1.698-4.380)	0.38
	ES	-0.077 (-4.808-4.655)	0.97	1.419 (-4.502-7.339)	0.64
L3/L4	MF	1.066 (-1.375-3.506)	0.39	1.968 (-0.973-4.910)	0.19
	ES	-0.282 (-3.846-3.281)	0.88	1.271 (-3.396-5.939)	0.59
L4/L5	MF	0.209 (-2.049-2.467)	0.86	0.721 (-2.068-3.510)	0.61
	ES	-0.972 (-3.209-1.265)	0.39	-0.758 (-3.588-2.071)	0.70
L5/S1	MF	-0.222 (-2.688-2.245)	0.86	0.162 (-2.892-3.217)	0.92
	ES	-0.058 (-1.695-1.580)	0.95	0.055 (-1.773-1.883)	0.95

FI= fat infiltration; MF= multifidus; ES= erector spinae; 95%CI = 95% Confidence Interval

Model 1 without adjusted

Model 2 adjusted for age, gender, BMI and intensity of cLBP

Supplemental Table 14. Effect size between muscle CSA, FCSA, FI and SF36.

		partial eta squared					
		CSA--SF36		FCSA--SF36		FI--SF36	
		left	right	left	right	left	right
L1/L2	MF	<0.01	<0.01	<0.01	<0.01	0.01	<0.01
	ES	<0.01	0.02	<0.01	0.02	<0.01	<0.01
L2/L3	MF	<0.01	<0.01	<0.01	<0.01	<0.01	<0.01
	ES	<0.01	0.04	<0.01	0.04	<0.01	<0.01
L3/L4	MF	0.04	0.03	0.02	0.02	0.01	<0.01
	ES	<0.01	<0.01	<0.01	<0.01	<0.01	<0.01
L4/L5	MF	0.04	0.03	0.03	0.02	<0.01	<0.01
	ES	<0.01	<0.01	<0.01	<0.01	<0.01	<0.01
L5/S1	MF	0.07	0.08	0.05	0.04	<0.01	<0.01
	ES	<0.01	<0.01	<0.01	<0.01	<0.01	<0.01

CSA= cross-sectional area; FCSA= functional cross-sectional area; FI= fat infiltration; MF= multifidus; ES= erector spinae
